# Oil-mediated high-throughput generation and sorting of water-in-water droplets

**DOI:** 10.1038/s41378-020-0180-0

**Published:** 2020-09-07

**Authors:** Lang Nan, Yang Cao, Shuai Yuan, Ho Cheung Shum

**Affiliations:** 1 Department of Mechanical Engineering, The University of Hong Kong, Pokfulam Road, Hong Kong, China; 2 Department of Electrical and Electronic Engineering, The University of Hong Kong, Pokfulam Road, Hong Kong, China

**Keywords:** Engineering, Materials science

## Abstract

Aqueous two-phase system (ATPS) droplets have demonstrated superior compatibility over conventional water-in-oil droplets for various biological assays. However, the ultralow interfacial tension hampers efficient and stable droplet generation, limiting further development and more extensive use of such approaches. Here, we present a simple strategy to employ oil as a transient medium for ATPS droplet generation. Two methods based on passive flow focusing and active pico-injection are demonstrated to generate water-water-oil double emulsions, achieving a high generation frequency of ~2.4 kHz. Through evaporation of the oil to break the double emulsions, the aqueous core can be released to form uniform-sized water-in-water droplets. Moreover, this technique can be used to fabricate aqueous microgels, and the introduction of the oil medium enables integration of droplet sorting to produce single-cell-laden hydrogels with a harvest rate of over 90%. We believe that the demonstrated high-throughput generation and sorting of ATPS droplets represent an important tool to advance droplet-based tissue engineering and single-cell analyses.

## Microfluidics: Employing oil medium to generate aqueous two-phase system droplets

Through using oil as a transient medium, a technique has been developed that can generate aqueous two-phase system (ATPS) droplets, thereby enhancing their application in various biological tests. Thanks to its ability to confine individual targets in microscale volumes, droplet microfluidics has great promise in processing and analyzing biological samples. Unfortunately, the ultra-low tension at the interface of ATPS (typically an aqueous solution of two incompatible polymers or one polymer and one salt) droplets has hampered their development for such applications despite their superiority over conventional water-in-oil droplets. However, a team headed by Ho Cheung (Anderson) Shum at the University of Hong Kong devised a simple strategy for using oil as a transient medium, which achieves a high throughput. The authors believe that their generation and sorting of ATPS droplets holds considerable potential for sophisticated microfluidic processing.

## Introduction

Droplet microfluidics has demonstrated vast promise in processing and analyzing biological samples due to its capacity to confine individual targets in microscale volumes and perform high-throughput manipulations^1–4^. However, the conventional use of water-oil droplet systems is constrained by the toxic nature of organic reagents, which reduces their biocompatibility and prevents access to droplet contents^5^. With an all-aqueous environment, water-in-water droplets produced by introducing aqueous two-phase systems (ATPSs) have attracted growing interest and shown great potential in fabricating biomimetic architectures^6,7^.

ATPS is typically an aqueous solution of two incompatible polymers or one polymer and one salt, which phase-separates to form two immiscible phases when the concentrations exceed their corresponding critical values^8^. The potential biocompatibility has promoted ATPSs as a promising candidate for various biological assays, including cell culture^9^, protein delivery^10^, and DNA partitioning^11^, but a strategy to generate water-in-water droplets is needed. To form droplets with uniform shapes and sizes, the two phase-separated immiscible phases are conventionally introduced into microfluidic channels for droplet formation^12^. However, interfaces of two aqueous phases can possess ultralow interfacial tension (typically in the range of 10^−4^ ~ 10^−1^ mN/m), challenging the efficient and stable generation of droplets in microfluidic systems: The droplets can be formed only under ultralow flow rates (below 3 μL/h) at frequencies below 10 Hz, and the droplet size distribution is also undesirably large^13^. Moreover, when polyelectrolytes or nanoparticles are added to stabilize the droplets, their fast assembly at the interface hampers pinch-off of the interface, further lowering the generation efficiency^14,15^.

To improve the droplet generation efficiency, active actuation strategies have been employed; by providing additional energy, pinch-off of the interfaces can be triggered. For instance, with the assistance of acoustic vibration and oil chopping to destabilize the jet, the generation frequency can be increased two-fold in comparison to passive generation in glass-capillary devices^16,17^. Polydimethylsiloxane (PDMS) devices with typically two-dimensional planar geometries are often used, where more sophisticated droplet manipulation is enabled, for instance, droplet sorting^18^ and pico-injection^19^. However, the shear force in these geometries dampens the active actuation, which becomes less effective in generating droplets with a high throughput^20^. As a result, even with mechanical actuation and eight parallel channels, the highest production rate is only approximately 100 Hz, which is still inefficient compared with water-in-oil droplet generation, typically with rates on the order of kHz^21^. Moreover, due to the similar electrical properties of the two aqueous phases, electrical manipulations of the generated droplets, such as dielectrophoretic (DEP)-based droplet sorting^22^, fail to work and preclude certain sample processing and analysis steps.

To address these problems, we propose an oil-mediated strategy to generate ATPS droplets, where water-water-oil double emulsions are first generated, with the inner aqueous cores subsequently released to form water-in-water droplets. To demonstrate the efficiency of this approach, double emulsions are passively generated in a flow-focusing channel at high throughput and with a uniform size distribution. The use of a transient oil medium also enables electric actuation to further increase the droplet generation rate and degree of control of the generated droplets. To extract the inner aqueous droplets, the transient oil layer is evaporated in a gentle and efficient manner. Through further incorporation of sodium alginate and integration of a droplet sorting system, single-cell-laden hydrogels can be produced, with a high harvest rate.

## Results

### Passive double-emulsion generation

**Fig. 1:**
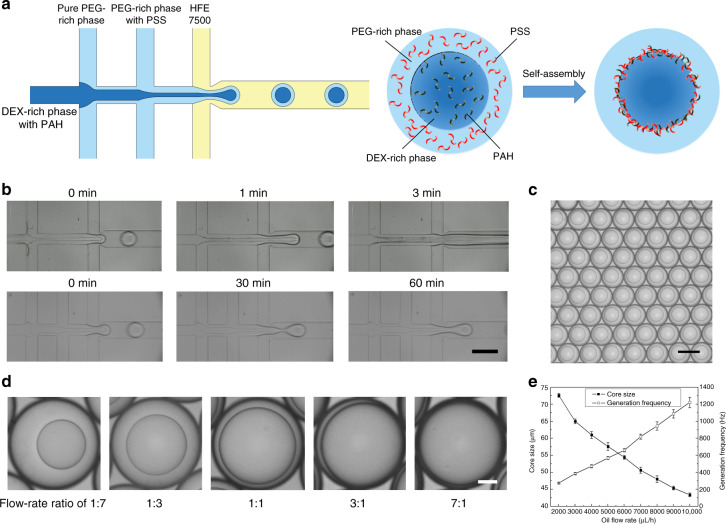
Passive generation of water-water-oil double emulsions. **a** Schematic of the passive method to generate water-water-oil double emulsions in a multi-inlet flow-focusing channel. **b** Comparison of the double-emulsion generation without and with a spacing stream. Scale bar: 200 μm. **c** Image of the double-emulsion drops generated at flow rates of 200 μL/h (DEX-rich), 600 μL/h (PEG-rich) and 3000 μL/h (oil). Scale bar: 100 μm. **d** The morphology of double-emulsion drops formed at different core-shell flow-rate ratios. The flow rate of oil is set constant at 3000 μL/h, while the flow rates of DEX-rich:PEG-rich phases are 100:700, 200:600, 400:400, 600:200 and 700:100 μL/h, respectively. Scale bar: 20 μm. **e** A plot of the core droplet diameter and generation frequency as the oil flow rate increases from 2000 to 10,000 μL/h

As the first step in ATPS droplet formation, the generation of water-water-oil double emulsions determines the final droplet size and morphology. To generate monodisperse double-emulsion drops, we passively break up the aqueous flow into droplets by oil pinching, which can be achieved in a typical multi-inlet flow-focusing channel^23^. Different from the water-oil-water (W-O-W) double-emulsion drops that are formed by two steps of emulsification^24^, water-water-oil (W-W-O) double-emulsion drops are formed in a single step at the second cross junction: The two aqueous phases flow laminarly side-by-side after meeting in the first junction, followed by pinch-off of the water–water compound jet by the oil flow to form W-W-O double-emulsion drops (Fig. 1a and Movie S1). To demonstrate the efficiency of this method, the two most commonly used ATPS polymers, polyethylene glycol (PEG) and dextran (DEX) that are both biocompatible, are introduced. To prevent coalescence and destabilization of the droplets during subsequent droplet extraction, two oppositely charged polyelectrolytes, poly(allylamine hydrochloride) (PAH) and poly(sodium 4-styrenesulfonate) (PSS), are dissolved into the innermost (DEX-rich) and intermediate (PEG-rich) solutions to stabilize the water-water interface through interfacial complexation. However, due to the fast reaction, premature precipitation prior to droplet breakup tends to gradually block the channel and cause a transition from the original dripping regime to an unstable jetting regime within 3 min. Thus, a spacing stream of pure PEG-rich solution is introduced at a flow rate of 100 μL/h to separate the two reagent streams, thereby enabling stable droplet generation for over 1 h and forming monodisperse double emulsions (Fig. 1b, c).

To optimize the microfluidic operations for controllable double-emulsion generation, the droplet size and generation frequency at different flow conditions are systematically studied. In this flow regime, the two streams of PEG-rich phases with and without PSS converge into one stream before droplet generation, and varying the flow rate ratio between the two PEG-rich phases does not lead to any observable effect on the resultant droplet size (Fig. S1). Thus, in the following analysis, we use the combined flow rate to characterize the flow behaviors. First, by regulating the flow rates of the aqueous phases, the formed core-shell structure can be controlled. We change the flow rate ratio of the DEX-rich phase and PEG-rich phase from 1:7 to 7:1 while keeping the oil flow rate constant. As a result, the inner droplet diameter increases while the outer droplet diameter remains unchanged, with the diameter ratio ranging from 50 to 96% and the resultant shell thickness varying from 26 μm to ~2 μm (Fig. 1d). The flow rate of oil is a key parameter that affects both the double-emulsion drop size and generation frequency. To quantify the effect of flow rate on the double-emulsion formation, we set the flow rates of DEX-rich and PEG-rich phases constant at 200 μL/h and 600 μL/h, respectively, while tuning the flow rate of oil from 2000 to 10,000 μL/h. For oil flow rates below 2000 μL/h, the aqueous compound jet cannot be pinched off. Experimentally, the core diameter gradually drops from 73 μm to ~43 μm as the oil flow rate increases, and the generation frequency increases from 270 Hz to ~1200 Hz (Fig. 1e). Since the two aqueous phases are formed by phase separation of PEG and DEX solutions, the adopted polymer concentrations determine the interfacial tension and viscosity of the two aqueous phases, thus affecting the interface pinch-off and droplet breakup. To understand the effect of polymer concentrations and test the feasibility of this approach in more viscous flows, we increase the PEG and DEX concentrations from 10 to 20% and measure the resultant core size and generation frequency at constant flow conditions (200 μL/h for the DEX-rich phase, 600 μL/h for the PEG-rich phase and 3000 μL/h for the oil phase). The results indicate that as the polymer concentration increases, the size of the core droplets increases from 65 μm to 77 μm, and the frequency decreases from 385 to 240 Hz (Tables 1 and 2). In summary, this method enables high-throughput generation of W-W-O double emulsions with controlled structures.

**Table 1:** The density, viscosity and interfacial tension of different concentrations of polymer solutions

Polymer solutions	Density (g/cm^3^)	Viscosity (Pa·s)	Interfacial tension (mN/m)
PEG-rich 10%	1.038	0.011	0.116
DEX-rich 10%	1.100	0.018	
PEG-rich 10%	1.036	0.014	0.157
DEX-rich 15%	1.130	0.030	
PEG-rich 10%	1.044	0.020	0.186
DEX-rich 20%	1.135	0.032	
PEG-rich 15%	1.039	0.018	0.173
DEX-rich 10%	1.127	0.033	
PEG-rich 15%	1.044	0.027	0.249
DEX-rich 15%	1.151	0.052	
PEG-rich 15%	1.048	0.034	0.394
DEX-rich 20%	1.165	0.072	
PEG-rich 20%	1.046	0.035	0.368
DEX-rich 10%	1.166	0.083	
PEG-rich 20%	1.050	0.044	0.478
DEX-rich 15%	1.179	0.123	
PEG-rich 20%	1.054	0.060	0.521
DEX-rich 20%	1.195	0.154

**Table 2:** The core-droplet diameter and generation frequency at different polymer concentrations in the passive generation mode

DEX-rich		M_w_ 8000 (PEG-rich)					
		10%		15%		20%	
		Core size (μm)	Frequency (Hz)	Core size (μm)	Frequency (Hz)	Core size (μm)	Frequency (Hz)
**M**_**w**_**10,000**	**10%**	65.0 ± 0.7	385.0 ± 10.6	66.6 ± 0.5	362.2 ± 7.1	73.4 ± 0.5	267.2 ± 3.8
	**15%**	68.2 ± 0.4	331.0 ± 4.5	72.2 ± 0.4	282.8 ± 4.4	74.6 ± 0.5	253.6 ± 3.3
	**20%**	70.2 ± 0.4	315.0 ± 4.5	74.4 ± 0.5	264.4 ± 3.1	76.6 ± 0.5	239.2 ± 2.7

### Active double-emulsion generation

**Fig. 2:**
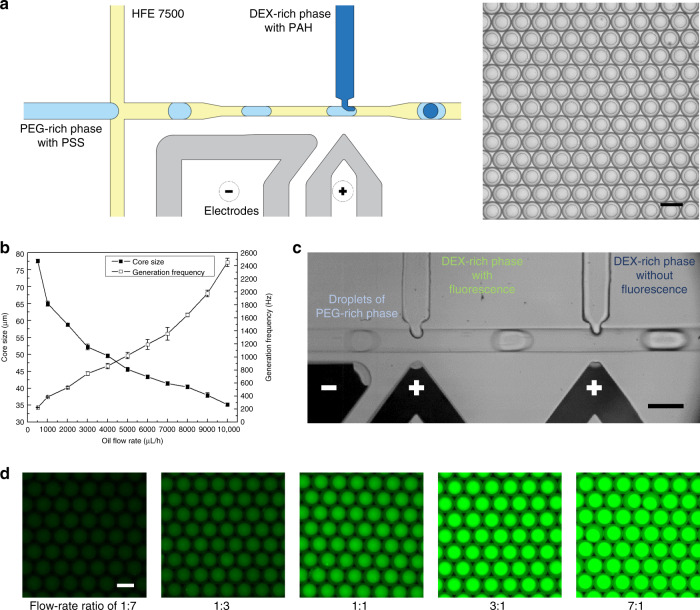
Active generation of water-water-oil double emulsions. **a** Schematic of the active method to generate water-water-oil double emulsions in a pico-injection channel and image of the double emulsions generated at flow rates of 200 μL/h (DEX-rich), 600 μL/h (PEG-rich) and 3000 μL/h (oil). Scale bar: 100 μm. **b** A plot of the core-droplet diameter and generation frequency as a function of the oil flow rate, which is varied from 500 μL/h to 10,000 μL/h. **c** Image of the dual-injector device for successive injection of the DEX-rich phases with and without fluorescence. Scale bar: 100 μm. **d** Fluorescence microscopy images of the resultant double emulsions at different flow-rate ratios of the two injections. The flow rates of PEG-rich and oil phases are set constant at 600 and 3000 μL/h, while the flow rates of the DEX-rich phases with and without fluorescence are coupled at 50:350, 100:300, 200:200, 300:100 and 350:50 μL/h, respectively. Scale bar: 100 μm

In addition to the demonstrated passive generation, double-emulsion drops can be actively generated by injecting the core droplet into the shell droplet one-by-one by pico-injection. Here, a typical pico-injection channel is employed for the two-step generation^25^. In the flow-focusing junction, the PEG-rich solution is broken up into droplets under the shear effect of the oil stream. Then, when the droplets flow by the pico-injector, a high voltage is applied to the electrodes to destabilize the water-oil interface, thus introducing the DEX-rich solution into the shell drop to form a double emulsion (Fig. 2a). This method relies on accurate control over the applied electric field, where an optimal pulse with a frequency of 10 kHz and peak-to-peak voltage of 500 V_p-p_ is applied to achieve a stable injection with identical volumes injected into each droplet^25^.

In comparison with the passive generation that simultaneously pinches off two aqueous streams, only one stream of the PEG-rich phase is segmented in the active operations, thus allowing more frequent double-emulsion generation. To demonstrate the higher efficiency, a similar test is performed to explore the effect of the oil flow rate on the double-emulsion generation. We change the flow rate of the oil phase from 500 to 10000 μL/h, while the flow rates of the droplet phase (PEG-rich) and injection phase (DEX-rich) are maintained at 600 and 200 μL/h, respectively. The results indicate that under the same flow conditions, smaller droplets can be generated at a higher frequency in the active mode. For instance, at the same oil flow rate of 3000 μL/h, the frequency of active generation (750 Hz) is approximately two times that of passive generation (380 Hz) (Fig. 2b). Moreover, double-emulsion formation at different polymer concentrations is studied. We set the flow rates of DEX-rich, PEG-rich and oil phases at 200, 600 and 3000 μL/h, respectively. Experimentally, when the concentrations of the two polymers increase from 10 to 20%, the resultant core size increases from 52 to 61 μm, and the generation frequency decreases from 750 to 480 Hz (Table 3).

**Table 3:** The core-droplet diameter and generation frequency at different polymer concentrations in the active generation mode

DEX-rich		M_w_ 8000 (PEG-rich)					
		10%		15%		20%	
		Core size (μm)	Frequency (Hz)	Core size (μm)	Frequency (Hz)	Core size (μm)	Frequency (Hz)
**M**_**w**_**10,000**	**10%**	52.2 ± 0.8	747.0 ± 30.1	54.6 ± 0.5	641.8 ± 23.0	56.0 ± 0.7	595.4 ± 16.5
	**15%**	54.4 ± 0.5	650.2 ± 23.0	56.2 ± 0.8	595.4 ± 16.5	58.6 ± 0.5	538.0 ± 16.4
	**20%**	57.2 ± 0.8	575.2 ± 17.5	59.2 ± 0.4	520.8 ± 11.6	60.6 ± 0.5	480.8 ± 10.7

In addition to the higher efficiency, the active generation method allows injection of different ingredients into the core droplets, thereby enabling time-controlled reactions within the droplets. We achieve this aim in a dual-injector device, where the two injectors are closely located at a spacing of 500 μm to efficiently merge the injected cores before the assembly of polyelectrolytes to form a membrane at the interface. To demonstrate the efficiency of this approach, a fluorescently labeled DEX-rich solution is introduced from the first injector, while a conventional solution without fluorescent dye is introduced from the second injector (Fig. 2c). As the flow rate ratio of the two injection flows is tuned from 1:7 to 7:1, double emulsions with different fluorescence intensities can be formed, achieving good mixing to allow accurate control of the fluorescent polyelectrolyte concentration (Fig. 2d).

### Core-droplet extraction

**Fig. 3:**
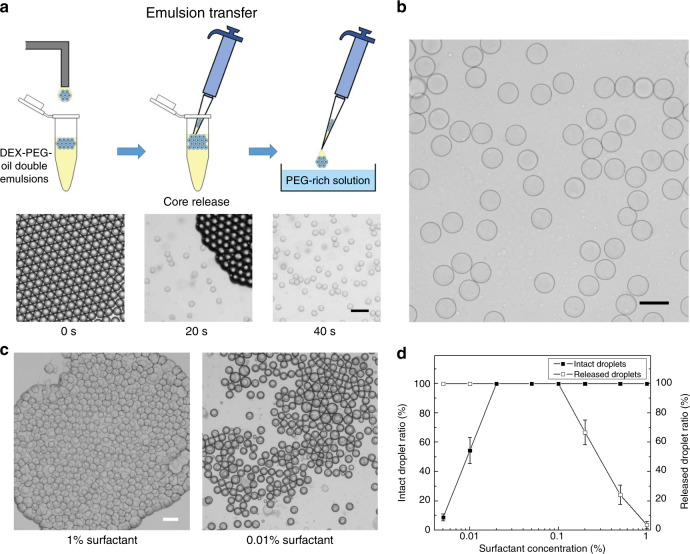
Extraction of the core droplets. **a** Schematic showing the process of transferring the double emulsions into a PEG-rich bath and images showing the process of releasing the inner core droplets from the double emulsions. Scale bar: 200 μm. **b** Image of the formed monodisperse ATPS droplets using the oil-mediated method. Scale bar: 100 μm. **c** Morphology of the double emulsion during oil evaporation at surfactant concentrations of 0.01 and 1%. Scale bar: 200 μm. **d** The chart showing the percentages of the droplets that are not coalesced and the droplets that are successfully released at different surfactant concentrations ranging from 0.005 to 1%

To extract the encapsulated core droplets for ATPS droplet formation, the carrier oil must be removed to break the double-emulsion drops. Typically, the droplets are broken by first adding the demulsifier to destabilize the surfactant layer and then centrifuging the samples to separate the water and oil phases based on their density difference^26^. However, this method involves severe flow disturbance, which tends to disrupt both the water-oil interface and water-water interface, thus inducing undesirable coalescence of the ATPS droplets. Therefore, gentler extraction should be employed to break the outer droplet interface while keeping the inner droplets intact. Here, we introduce a noninvasive method that hinges on evaporation of the oil phase to break the double emulsions. This method is performed by transferring the collected emulsions into a Petri dish filled with the PEG-rich solution. When the emulsions fall onto the solution, the interfacial tension keeps the oil phase on the top, with the double-emulsion drops aggregating at the center. As the surrounding oil spreads and forms a thin layer, its surface area increases. Consequently, the oil layer evaporates in ~20 s, and the shell phase merges with the PEG-rich solution underneath; hence, the cores of the DEX-rich phase are released to form monodisperse DEX-in-PEG droplets (Fig. 3a, b).

In this method, the surfactant concentration is a key factor in implementing core-droplet extraction. To elucidate the effect of the surfactant concentration on the extraction efficiency, droplets generated using different concentrations of surfactant, ranging from 0.005 to 1%, are tested. The results indicate that when the surfactant concentration is below 0.02%, droplet coalescence occurs due to the unstable water-oil interface; when the surfactant concentration is above 0.2%, the droplets remain separated from the PEG-rich solution after complete oil evaporation (Fig. 3c, d). Thus, the surfactant concentration should be kept at appropriate ranges to stabilize the interface and release the core droplets. Using 0.05% surfactant as a demonstration, the core droplets can be efficiently extracted within 40 s. Moreover, with this evaporation-driven extraction, the inner core droplets are free from external disturbance and can maintain their uniform shapes and sizes (coefficient of variation of approximately 2%).

### Single-cell-laden microgel sorting

**Fig. 4:**
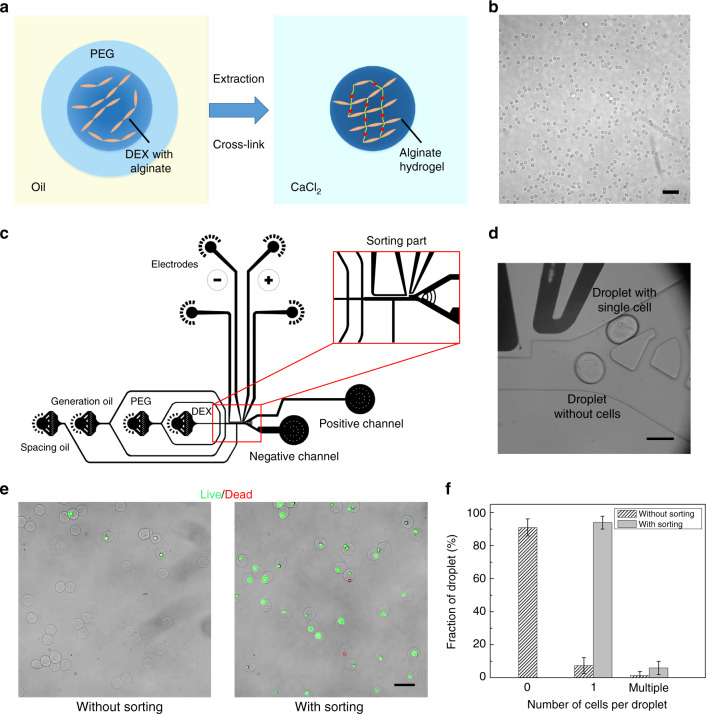
Fabrication of single-cell-laden hydrogels. **a** Schematic showing the process of crosslinking the alginate solution by Ca^2+^ diffusion. **b** Image of the formed monodisperse alginate microgels. Scale bar: 200 μm. **c** Design of the integrated channel to perform continuous droplet generation and sorting. **d** Image illustrating the sorting process: The droplets with single cells are driven to the positive channel, while the droplets without cells flow into the negative channel. Scale bar: 100 μm. **e** Comparison of the formed hydrogels without and with sorting after a Live/Dead assay. Scale bar: 100 μm. **f** A plot of the percentages of the droplets encapsulating zero, single and multiple cells without and with sorting

Droplet-based hydrogel microspheres, which can provide both mechanical stability and cell-binding properties, are promising scaffolds for 3D cell culture and single-cell analysis^27,28^. However, the conventional fabrication method based on a water-oil system requires strong mechanical and chemical treatment to extract the formed hydrogels from organic solvents, possibly resulting in loss of cell viability^29^. In comparison, the demonstrated ATPS droplet facilitates biocompatible microgel fabrication in an all-aqueous environment. We achieve this aim by incorporating alginate, a biocompatible polymer that can be physically crosslinked with divalent ions^30^, into the DEX-rich phase. Through collecting the double emulsions into a calcium chloride (CaCl_2_) solution bath, the released core droplets can be rapidly crosslinked to form spherical and uniform-sized hydrogels (Fig. 4a, b).

Moreover, the transient use of the oil phase enables inclusion of a droplet sorting step after double-emulsion generation and before extraction of the core droplets to produce single-cell-laden hydrogels, which is difficult to achieve in other ATPS droplet generation approaches^31^. We demonstrate this process in an integrated channel composed of both generation and sorting parts (Fig. 4c). Here, a spacing oil flow is introduced to further gap the continuously generated droplets for accurate sorting, and the two branch channels are designed with precise differences in hydraulic resistances. As a result, the droplets are guided into the lower channel when no electric field is applied and are driven into the upper channel when an electric field is applied (Fig. 4d). Through setting the flow rates of the core, shell, generation and spacing oil phases at 200, 600, 3000 and 3000 μL/h, respectively, high-throughput sorting can be achieved at frequencies above 300 Hz. To demonstrate the sorting-based fabrication of single-cell-laden microgels, the average cell number in each droplet is tuned to 0.1 so that only approximately 10% of the generated droplets contain single cells following the Poisson distribution. With a sorting step to remove the negative droplets, the single-cell encapsulation ratio increases to over 90%, thus allowing a high-throughput analysis of the encapsulated single cells (Fig. 4e, f). Moreover, to verify the biocompatibility of this technique, a live/dead assay is performed on the encapsulated cells and shows high viability, with over 95% of the cells remaining alive. This result suggests that the encapsulated cells can be further cultured for tissue development and engineering applications.

## Discussion

The core concept of this technique is to first use oil as a continuous phase to rapidly generate W-W-O double-emulsion drops and then remove the oil to release the inner cores for water-in-water droplet formation. With the use of oil as a continuous phase, sorting can be further incorporated after the formation of double-emulsion drops and before the extraction of core droplets to select the candidates of interest.

In double-emulsion generation, the low interfacial tension that is frequently encountered for water-in-water droplets can be overcome by introducing an oil phase, thus achieving high levels of throughput and monodispersity in both passive and active methods. Through adjusting the flow rates, the morphology of the double-emulsion drops formed can be precisely controlled, and the generation frequency can be as high as ~2.4 kHz, which is 20 times higher than that of existing methods. Moreover, the fabricated double-emulsion drops can be used to form ATPS microcapsules by adding hydrogel components into the shell phase, which may serve as biocompatible carriers in drug delivery and food processing assays^32^.

The approach of active double-emulsion formation through sequential droplet injection is efficient for performing controlled reactions within the inner droplets, which is not limited to the demonstrated sample mixing and can be extended to other functional operations. For instance, through the incorporation of different individual cells by sequential injections, cell-to-cell communications and interactions at the single-cell level can be studied within biocompatible compartments. Moreover, if we further increase the gap between each injector or perform sequential injections in separate devices, the core droplet can be stabilized before subsequent injection, thus forming double emulsions with multiple separate cores. This type of structure is potentially applied in fabricating cell-like biomimetic architectures, such as protocells, where the core droplets can function as independent artificial organelles.

To gently break the double emulsions without damaging the inner cores, an oil evaporation-based method is introduced, enabling fabrication of monodisperse ATPS droplets with spherical shapes. The obtained all-aqueous microspheres can serve as biocompatible templates to perform various assays, as demonstrated in the microgel particle fabrication and DNA partitioning studies. Moreover, the employed core-release method is useful for recovering single-emulsion drops, which can be functionalized to extract the encapsulated single cells from water-in-oil droplets^4^.

When combined with accurate droplet sorting, the demonstrated production of single-cell-laden hydrogels promises further high-throughput analysis of the encapsulated single cells, such as detecting their immune responses to study the distribution of antibiotic resistance and monitoring their differentiation to probe the heterogeneity among populations. Moreover, noninvasive operations guarantee high cell viability, enabling further culture to develop microgels into functional building blocks for bottom-up tissue engineering^33^.

Despite the high efficiency of this method in generating and sorting ATPS droplets, there is still room to further exploit its potential. For instance, the finally formed droplets are still in a single-emulsion format, which restricts their use in applications that require controlled sample encapsulation and release^34^. Thus, further development should focus on the fabrication of more functional aqueous multiple-emulsion drops, which may be achieved by the introduction of additional immiscible aqueous flows for compartmentation.

In summary, the introduction of a transient oil medium enables high-throughput generation and sorting of water-in-water droplets, which can be used as templates to encapsulate single cells in hydrogel microspheres. The simplicity, high throughput and capacity to be integrated into more sophisticated microfluidic processes inspire new ways to use these droplets in various material synthesis and biological analyses.

## Materials and methods

### ATPS droplet generation

To prepare ATPS solutions, PEG (M_w_ 8000, Sigma-Aldrich, USA) and DEX (M_w_ 10,000, Sigma-Aldrich, USA) polymers were dissolved in deionized water at weight ratios from 10 to 20%. After centrifugation at 8000 rpm for 1 h, the solutions were left standing overnight to achieve complete separation. Then, the equilibrated PEG-rich and DEX-rich solutions were collected separately, and the polyelectrolytes, PSS and PAH (Sigma-Aldrich, USA), were dissolved into the two phases at concentrations of 2 mg/mL. For further fluorescent labeling in the sequential injection studies, fluorescein isothiocyanate-dextran (M_w_ 10,000, Sigma-Aldrich, USA) was dissolved into the DEX-rich phase at a weight ratio of 0.5%. A fluorinated oil (HFE7500, 3M, USA) was used as the continuous phase for droplet generation, and the weight ratios of a fluorosurfactant (008, RAN Biotechnologies, USA) were varied from 0.005 to 1%. To form alginate hydrogels, an alginic acid sodium salt from brown algae (low viscosity, Sigma-Aldrich, USA) was dissolved into the DEX-rich phase at a weight ratio of 1%, and a 1% weight ratio of CaCl_2_ solution (Aladdin, CN) was used to extract the core droplets.

### Device fabrication

The microfluidic devices were fabricated using a typical soft lithography replica molding technique^35^. First, a channel mold was fabricated on a silicon wafer (N100, University, USA) with SU-8 photoresist (2025, MicroChem, USA) using maskless lithography (SF-100 Xcel, Intelligent Micro Patterning, LLC, USA). Then, the PDMS prepolymer base (Sylgard 184, Dow Corning, USA) was crosslinked with the curing agent at a weight ratio of 10:1. After sufficient mixing by a conditioning mixer (AR-100, THINKY, JP), the mixture was poured onto a channel mold and cured at 65 °C for 4 h. Subsequently, the PDMS channel was peeled off from the mold. Finally, after inlet and outlet punching, the PDMS channel was bonded to a glass substrate (ISOLAB, DE) through oxygen plasma treatment (PDC-002, Harrick, USA) and heated at 90 °C for 2 h. The height, width and length of the generation channel were designed to be 65, 120 and 2000 μm, respectively, and the whole channel was treated with a hydrophobic agent (Aquapel, PPG, USA) to guarantee stable droplet generation. To fabricate the electrodes required in active generation and sorting, empty channels were fabricated in the desired shape of the electrodes. Then, a low-melting-point metal wire (52225, Indium Incorporation, USA) was inserted into the electrode channel from one end at 95 °C, and negative pressure was applied to the other end to fill the whole channel with liquid metal. After further cooling the device to solidify the metal, metallic electrodes could be formed according to the desired shape.

### Cell assay

An esophageal cancer cell line (KYSE-150) was used as the target encapsulant to test the sorting efficiency. For fluorescence labeling, the cells were transduced with lentiviral particles encoding green fluorescent protein (GFP). The coding sequence was cloned into a pLVX-puro backbone (Clontech, USA) following an EF-2 alpha promoter^36^. The cells were maintained in Roswell Park Memorial Institute (RPMI) 1640 Medium (Life Technologies, USA), 100 U/mL penicillin and 100 μg/mL streptomycin (Gibco, USA) in a humidified atmosphere at 37 °C with 5% CO_2_. After trypsin treatment, the cells were transferred into the prepared DEX-alginate solution at a concentration of 3.05 × 10^6^/mL. The cell viability after encapsulation and sorting was tested using a Live/Dead reagent (calcein AM/ethidium homodimer-1, Sigma-Aldrich, USA).

### Droplet sorting

A typical florescence-activated droplet sorting (FADS) system was used to sort the double-emulsion drops with single cells^37,38^. A diode-pumped solid-state (DPSS) laser source (20 mW, CNI, CN), with a wavelength of 488 nm, was used to excite the fluorescence signal. Guided by a dichroic mirror (Semrock, USA), the laser beam was reflected into a plan fluorite objective (Olympus, JP) and focused into the microfluidic channel. Then, the excited fluorescence signal propagated backward and was separated into two beams through a beam splitter (Thorlabs, USA), with 90% of the light received by a photomultiplier tube (PMT, Hamamatsu, JP) for fluorescence detection and the remaining 10% used for high-speed imaging (Phantom V9.1, Vision Research, USA). Subsequently, the triggered signal was processed by a custom LabVIEW program (PCI-e 7842R, National Instruments, USA), responding with output signals applied to the electrodes after amplification by a high-voltage amplifier (5/80, Trek, USA).

## Supplementary information

Movie. S1

Movie. S2

Movie. S3

Movie. S4

Supplementary Information
