# Supplementary material for: Oil-mediated high-throughput generation and sorting of water-in-water droplets
Source: Microsyst Nanoeng. 2020 Sep 7;6:70. doi: 10.1038/s41378-020-0180-0 (PMC8433215; doi:10.1038/s41378-020-0180-0)
Supplement: Supplementary file 5 — Supplementary Information [file 41378_2020_180_MOESM5_ESM.docx]

**Supplementary Information**

**Title:** Oil-mediated high-throughput generation and sorting of water-in-water droplets.

Lang Nan, Department of Mechanical Engineering, The University of Hong Kong, Pokfulam Road, Hong Kong, China

Yang Cao, Department of Mechanical Engineering, The University of Hong Kong, Pokfulam Road, Hong Kong, China

Shuai Yuan, Department of Electrical and Electronic Engineering, The University of Hong Kong, Pokfulam Road, Hong Kong, China

Ho Cheung Shum, Department of Mechanical Engineering, The University of Hong Kong, Pokfulam Road, Hong Kong, China, Telephone: (852) 39177904, Email: [ashum@hku.hk](mailto:ashum@hku.hk)

These authors contributed equally: Lang Nan and Yang Cao


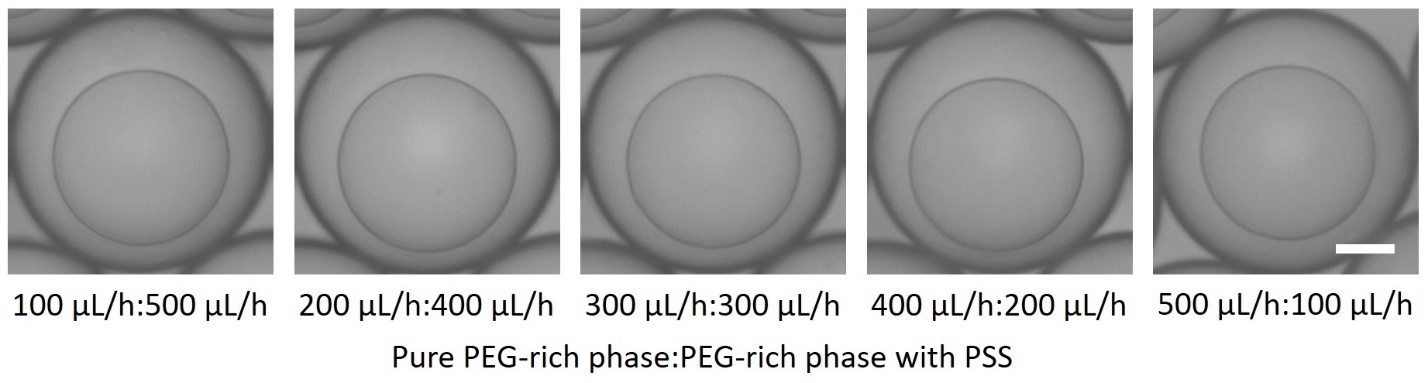


**Figure S1.** The size of the formed double-emulsion drops remains the same for different flow rate ratios of the pure PEG-rich phase and PEG-rich phase with PSS. The flow rates of the DEX-rich phase and oil phase are set constant at 200 μL/h and 3000 μL/h. Scale bar: 20 μm.

**Movie S1.** Passive generation of DEX-PEG-oil double emulsions in a flow-focusing channel at flow rates of 200 μL/h (DEX-rich phase), 600 μL/h (PEG-rich phase) and 3000 μL/h (oil phase). The movie is recorded at 1000 fps and played at 10 fps.

**Movie S2.** Active generation of DEX-PEG-oil double emulsions in a pico-injection channel at flow rates of 200 μL/h (DEX-rich phase), 600 μL/h (PEG-rich phase) and 3000 μL/h (oil phase). The movie is recorded at 1000 fps and played at 10 fps.

**Movie S3.** Release of the inner cores from double emulsions to form monodisperse water-in-water droplets through oil evaporation. The movie is played at normal speed.

**Movie S4.** Fluorescence-activated sorting of the double emulsions encapsulating single cells. The movie is recorded at 1000 fps and played at 10 fps.
